# Turning nursing students’ mistakes into resources for learning in simulation-based training: facilitators’ assumptions about providing feedback in debriefing

**DOI:** 10.1186/s12909-024-06628-z

**Published:** 2025-01-16

**Authors:** Wenche Lervik, Mads Solberg, Astrid Camilla Wiig, Helen Berg

**Affiliations:** 1 Department of Health Sciences, Norwegian University of Science and Technology, Ålesund, 6009 Norway; 2 Department of Educational Science, University of South-Eastern Norway, Notodden, Norway

**Keywords:** Simulation-based training, Simulation-based learning, Facilitator, Feedback, Debriefing, Assumptions, Experiences, Nursing-students

## Abstract

**Background:**

The aim of this study was to investigate how facilitators approach and use nursing students’ mistakes in simulation-based training as learning resources in the simulation debriefing phase. Facilitators are responsible for raising students’ awareness of their performances during the debriefing and facilitating reflections on their performances, including satisfactory behaviours and performance gaps. Research on facilitators’ work during debriefing has highlighted various challenges, such as providing a safe and constructive climate among novice students while simultaneously teaching them the correct procedures, methods, and knowledge of caring practices to become professional nurses. There is a lack of research on how facilitators approach, handle, and use students’ mistakes as a learning resource. Thus, this study investigated facilitators’ assumptions about providing feedback to nursing students when they made mistakes during simulation-based training

**Method:**

Individual semi-structured interviews were conducted with nine experienced facilitators from three universities in Norway. Data were analyzed following the principles of thematic analysis (TA).

**Results:**

Facilitators made varying assumptions about the simulations and debriefings as learning processes. These differences were evident in their accounts of how feedback was provided to students when they made mistakes during the simulation-based training.

**Conclusion:**

Facilitators’ statements about their practices reflect assumptions about how they make simulation activities a resource for meaningful learning, including how to use students’ mistakes as learning opportunities during debriefing discussions. Consequently, these assumptions regarding learning provide valuable insights into the ambiguous and complex praxis of using simulation-based training as a professional educational tool.

**Supplementary Information:**

The online version contains supplementary material available at 10.1186/s12909-024-06628-z.

## Background

Mistakes occur in all spheres of life; however, in healthcare services, mistakes can cause adverse events with serious consequences for the patient, next of kin, health professionals, and institutions [5]. In simulation settings, mistakes are sometimes considered learning opportunities that would otherwise put the patients at risk [6].

In their review of student perceptions and experiences of errors in simulations, Palominos et al. [7] described how mistakes can affect student learning. They found that even though some students felt negative about making mistakes in simulations, certain elements, such as a safe learning space where experienced facilitators provided constructive feedback and an environment where students were encouraged to discuss their mistakes, helped mitigate these negative feelings, potentially turning mistakes into opportunities for learning. However, to maximize learning from mistakes, facilitators must intentionally plan and assist students in identifying, admitting, and effectively addressing these mistakes [7].

Effective simulation-based training in nursing education relies on a skilled facilitator, often a teacher, who possesses the necessary education, expertise, motivation, and ability to guide and support students as they work toward achieving specific learning objectives. According to Persico et al. [8], the facilitator’s role involves creating and delivering simulations, defining clear learning objectives, selecting an appropriate level of simulator fidelity, and skillfully leading students through briefings, scenarios, discussions, and reflections during debriefing. To ensure optimal learning achievement, facilitators play a crucial role in raising students’ awareness of mistakes and shortcomings in their performances compared to professional standards [9, 10].

Research has emphasized that the debriefing phase is pivotal in simulation-based training [11, 12]. During debriefing, participants are encouraged to discuss their performance strengths and weaknesses [13]. Here, students receive valuable feedback from facilitators or peers, engage in thoughtful discussions, and reflect on their performances [14, 15].

The feedback phenomenon is well described in educational literature [15–18]. In a highly influential review by Hattie and Timperley [19], feedback is conceptualized as information provided by an agent (teacher, peer students, parent, book, self, or others) about how an action was performed. Feedback can be delivered in multiple ways: verbally, as corrective advice from teachers or peers, or through written resources that explain students’ performances. Both verbal and written feedback typically involves comparing the performance with an ideal standard. Hattie and Timperley [19] suggest that feedback is one of the most potent influences on learning achievement, with its impact being either positive or negative, depending on how it is provided. For feedback to have an effect, Hattie and Timperley [19] argue that there must be a learning context in which input is provided after students have responded to the learning instruction.

Most facilitators consider a safe environment essential for learning because it can decrease students’ stress and anxiety [20, 21]. Rudolph et al. [22] refer to a safe environment as a “safe container”. In such an environment, students can perform without fearing negative consequences or reprimands for mistakes. When students feel safe, they can extend their comfort zone, reflect on and discuss areas needing improvement, and make mistakes without feeling ashamed [23].

Despite regularly assuring students that there are no penalties for making mistakes and emphasizing that mistakes should be viewed as valuable learning opportunities, many facilitators still experience serious dilemmas when providing feedback during debriefing [16, 24].

In their review of debriefing methods, Sawyer et al. [25] describe feedback as a process intended to alter students’ thought processes and actions, thereby enhancing learning through a two-way, interactive, and reflective discussion between the facilitator and students in a team setting. In the reflective discussion during debriefing, facilitators should be aware of their basic assumptions regarding students’ motivation, interest in simulation-based training, and capabilities to participate in the debriefing effectively.

Lymer and Sjöblom [26] documented that simulation-based research on debriefing is dominated by quantitative studies examining learning outcomes, efficiency, best practices and the factors that moderate its effectiveness. Despite extensive qualitative research on debriefing and feedback, few studies have investigated facilitators’ fundamental assumptions and beliefs when providing feedback, especially when students make mistakes. Given that these assumptions likely shape debriefing practices, this study aimed to investigate how facilitators approach and use nursing students’ mistakes in simulation-based training as learning resources in the simulation debriefing phase.

## Methods

This qualitative study used thematic analysis drawing on semi-structured individual interviews with individuals based on open-ended questions.

### Setting and recruitment

In the autumn of 2022, the first author visited three universities in Norway and observed high-fidelity simulation-based training for nursing students in their second year of education. A range of diagnoses with similar complexity and aligned learning objectives was conducted across the three universities. All simulation sessions were normatively assessed. At one of the universities, they used the PEARLS framework during debriefings [27]. The two other universities did not use any formal framework. A total of 17 facilitators guided the students through these simulations. The study aimed to sample the most experienced and educated facilitators in clinical practice and simulation-based training. It was assumed that these experienced facilitators had encountered numerous challenging debriefing situations during their practice. Ten facilitators met the criteria. These ten facilitators were contacted via email or phone with an inquiry about whether they would like to participate in an interview study.

### Participants

Nine facilitators provided written informed consent to participate in the study. Facilitators from all the universities were represented, three men and six women. All the participants were registered nurses, and four had continued education as intensive care nurses.

Four were lecturers, and five were associate professors; they had diverse teaching backgrounds spanning 2–26 years of teaching experience. All participants regularly facilitated nursing students in simulation-based training and completed formal facilitator training courses, with facilitator experience varying from 6 to 11 years (Table 1 NEAR HERE).

**Table 1:** Participants’ demographic data

Participant	Gender	Profession	Teaching experience	Facilitator experience
*P1*	*M*	*Nurse/Lecturer*	*2 years*	*10 years (clinic)*
*P2*	*W*	*Intensive care nurse/Professor*	*26 years*	*11 years*
*P3*	*M*	*Intensive care nurse/Lecturer*	*4 years*	*10 years (clinic)*
*P4*	*W*	*Intensive care nurse/Professor*	*25 years*	*10 years*
*P5*	*W*	*Intensive care nurse/Associate professor*	*10 years*	*8 years*
*P6*	*W*	*Intensive care nurse/Associate professor*	*11 years*	*9 years*
*P7*	*M*	*Intensive care nurse/Associate professor*	*8 years*	*8 years*
*P8*	*W*	*Nurse/Lecturer*	*8 years*	*6 years*
*P9*	*W*	*Nurse/Lecturer*	*12 years*	*8 years*
		*Mean*:	*10*,*66 (11)*	*8*,*88 (9)*

### Data collection

The data were collected between January and April 2023. The first author conducted the interviews online, on Teams digital platform. Interviews were recorded using the Teams integrated recording feature, based on participants’ consent. The authors developed a semi-structured interview guide for the study (see Additional file 1). The interview guide was based on previous studies probing issues such as facilitators’ responsibilities and their attitudes toward providing feedback [16, 28, 29]. The guide comprises four topics. The first part included general questions about facilitators’ experiences with and views on simulation-based training. The second part examined facilitators’ perspectives on providing feedback when students made mistakes. The third topic reviewed the facilitators’ use of structured frameworks during the debriefing. The fourth and last set of questions examined the facilitators’ views of the atmosphere in the debriefing room. To ensure the relevance and comprehensibility of the questions, two pilot interviews were conducted with facilitator colleagues to refine and revise the interview guidelines. The interviews lasted from 23 to 70 min. All participants were informed about the study’s aim, personal data management, and their right to withdraw without consequences. They provided written consent, and the data from each interview was handled in line with relevant ethical and privacy guidelines and regulations. The participants’ anonymity was maintained throughout the process using gender-neutral language and pseudonyms when presenting quotes.

### Data analysis

Data were analyzed following the six phases of thematic analysis (TA) inspired by Braun and Clarke [28]. Initially, the first author manually transcribed the digital recordings by repeatedly listening to the audio. NVivo Software (version 12.0) was used to code and organize the data, yielding 350 codes. All authors met several times to review and discuss the initial themes. Codes with shared meanings were clustered into groups based on their relevance to the research objectives and jointly reviewed. Some clusters were deemed relevant to the study, while others were omitted. Initial themes were created by identifying data patterns related to how facilitators described the concept of mistakes and feedback, and their perspectives on addressing mistakes. Initially, the authors agreed on eight identified themes. By compiling clusters of codes, broader meanings emerged based on the constant movement between codes, initial themes, and transcripts. Eventually, four themes were refined and reviewed by all the authors and peer-checked by an extended group of nursing researchers with experience in simulation-based training. (Table 2 NEAR HERE).

**Table 2:** Experpts from analysis 281024

Quotation	Code	Initial theme	Theme
“It seems difficult to connect with them, they are incredibly stressed”	A high level of stress prevents connection	Efforts to decrease stress are required	Creating a safe place for learning
“Here you are allowed to make mistakes, this is no exam, we have not gathered you [here] to test you on anything.”	Students can make mistakes with no consequences	Creating an environment where the facilitator can discuss the performance, both the satisfactory behaviours and areas that need improvements	Creating a safe place for learning
“I believe we have a responsibility and need to focus on it [safe environment]. Sometimes it’s very easy because the students do great work. Other times, it can be challenging when some students mess things up, but I just have to say it”	A safe environment is essential in both challenging simulation situations and those that go well	Making an environment where both facilitators and students can communicate about the positive and the things that need improvement	Creating a safe place for learning
“As a rule, they see all the mistakes themselves, they just mention them themselves. I can never mention anything negative, because they see it themselves.”	The facilitator assumes that students are aware of their mistakes	The facilitator has a strong belief in students’ abilities to understand and make meaning of their mistakes	Variability in facilitators’ responses when addressing students’ mistakes
“I am very concerned that they receive positive feedback. Everyone must get something positive back. One must address those things first and focus on what they did well.”	Emphasizing the positive first	Positive feedback provides a sense of mastery	Fostering positive aspects to enhance learning

## Results

In this study, we focus on four themes developed from an analysis of the empirical material. The first theme concerns how the facilitators reflect on the concept of making mistakes. The second addresses how facilitators emphasize the positive aspects of simulation events to enhance learning. The third theme outlines facilitators’ concerns about creating a safe place for learning. The fourth theme revolves around how the facilitators use a variety of approaches to address mistakes.

### How facilitators talked about mistakes

When the facilitators discussed mistakes as phenomena, they did not specify or define what they considered a mistake. Instead, they alternated between expressions like making “severe mistakes” and “minor mistakes” when discussing mistakes in general. Making minor mistakes was not further explicated or clarified as a concept but when they explained how students were making severe mistakes, several informants used incorrect handling and dosage of medications as a typical example. On four occasions, three facilitators used the metaphor “the elephant in the room” when discussing serious mistakes, often related to medication management. One of the facilitators expressed it like this: “Certainly, if it’s big things, like if there’s a big elephant in the room, such as being given a ten-fold dose of adrenaline, for example, then you just have to discuss it.” (Nancy).

Some facilitators exemplified other severe mistakes by outlining previous situations in which students’ lack of knowledge and competencies made them unable to assess the patient’s vital values or detect the patient’s deterioration in time. Other mistakes were deemed so severe that they posed a risk of harm or fatality to patients. One other facilitator said: “If it was a very serious mistake so that the patient could have died, [only] then I bring it up. I have never experienced it, though, but I would have brought it up.” (Jack).

Most facilitators consider that making mistakes during simulations can provide valuable learning experiences for students. Some consider it an advantage if the students make mistakes during the simulations because it provides opportunities to use the example to discuss possible alternatives for their actions and behaviours. Thus, while reflecting on mistakes as a phenomenon, facilitators relate these to the performance of satisfactory procedures.

### Fostering positive aspects to enhance learning

Several facilitators highlighted positive feedback as beneficial to learning when students performed in a simulated environment. Some believed that the students should receive a positive comment to instill the feeling of performing well as a nurse in an “as-if” situation, regardless of their overall practice. One of the most experienced facilitators said:

I am very concerned that they receive positive feedback. Everyone must get something positive back. One must address those things first and focus on what they did well, and it applies to me as a facilitator, and it applies to the observers. (Mary)

It seems like providing a positive environment or climate for engaging in a simulated situation was important for both the facilitator and students acting as well as observers so that all students could feel confident in acting “as if” they were in a professional practice.

In addition to fostering positivity, some facilitators said that they felt it was important that the students were aware of the correct behaviours and acted in line with the relevant procedures when they completed the simulations. They considered the simulation area a safe place to make mistakes and argued that students could reflect on, discuss alternatives, and learn from their misconceptions in this supportive environment. One facilitator articulated the issue as follows: “One will always highlight things that are positive and start with them. At the same time, I also think it is important that one focuses on the things that could have been done differently.” (Chris).

Furthermore, some facilitators said they considered it important that the students were able to admit their mistakes, and that they were able to recognize the consequences the mistakes could lead to in the clinic.

Certain facilitators expressed their desire for students to achieve a sense of mastery during simulation activities. They viewed this sense of mastery as their responsibility when facilitating the simulation. One facilitator linked students’ mastery to their potential for learning and stated that the mastery of performance in the simulation could enhance students’ self-esteem. Another facilitator said they considered the students’ sense of mastery positively influencing their future roles as professional nurses. In the words of an experienced facilitator:

I have often experienced that they are extremely nervous about giving injections, but they attend anyway. They are so nervous, they vomit on the floor, screaming, and are completely distraught. Then comes the question: How do I calm them down? How do I get them to cooperate? How do I help them through this? This is also part of facilitation… How do I make them feel a sense of mastery despite a bad starting point? I always find that exciting. (Claire)

Guiding nervous students through the first performative actions seems to be a pivotal and exciting responsibility for facilitators, as they are responsible for raising professionalism and creating scaffolding activities during students’ learning trajectories.

Occasionally, facilitators perceived students as being overly critical of their performances. For instance, when encountering self-critical students who underestimated themselves, they described how students or their peers would be encouraged to highlight the positive aspects of their performances. This was expressed as follows: “I ensure they avoid excessively underestimating themselves, as this is a common occurrence, it’s quite clear that it’s crucial to refrain from undermining oneself.” (Susan).

By focusing on the positive aspects of simulation performances, facilitators aimed to ensure that students left the simulation-based training with a sense of proficiency.

### Creating a safe space for learning

All the facilitators interviewed described how they considered a safe atmosphere essential in simulator-based training. In their experiences, most students were stressed and anxious when they participated as nurses or observers in simulation scenarios and debriefings. By rendering the simulation situation safe and emphasizing that students would not be held accountable for any mistakes, they attempted to create a safe learning environment. The facilitators agreed that simulations were stressful situations that could prevent students from following the correct procedures and guidelines. Hence, facilitators put significant effort into creating safe environments. One facilitator expressed a sense of responsibility as follows:

I believe we have a responsibility and need to focus on it [safe environment]. Sometimes it’s very easy because the students do great work. Other times, it can be challenging when some students mess things up, but I just have to say it. (Betty)

Some facilitators described their efforts to establish trust by politely presenting themselves and clearly explaining the learning objectives when they met the students in the initial briefing. Others aimed to create a friendly atmosphere by sharing stories from their practice. The facilitators assumed that recounting their past mistakes helped students feel safer and more relaxed. Some said they used humor and laughter to ease students’ stress and tension. One of the facilitators used to explicitly discuss stress and anxiety during the briefing because they knew that these emotions were common among the students. Others said they used to assure the students that the simulation was not a test but a training area where they were supposed to practice and learn from mistakes, contrary to an ordinary exam where they could pass or fail.

One of the facilitators advised students to reduce their stress and anxiety by telling them: “Here you are allowed to make mistakes, this is no exam, we have not gathered you [here] to test you on anything.” (Mike).

One way in which the facilitators attempted to create a safe environment was to ensure confidentiality within teams. Typically, facilitators would inform the students that “the events within these walls remain within these walls,” thereby emphasizing that students should not discuss their peers’ actions and behaviours with other students outside the simulation area. Additionally, the facilitators enforced a policy prohibiting students from taking photos or recording videos during the simulation. They believed that if students felt secure, they would be encouraged to behave more freely and openly, exposing their vulnerabilities to facilitators and peers.

### Variability in facilitators’ responses when addressing mistakes made by students

Some facilitators believed that mistakes should be openly discussed within the group. A few argued that their goal was to educate highly qualified professional nurses who could distinguish between right and wrong when they entered the clinic. Some described an approach in which they initiated a dialogue by highlighting a mistake or asking students if they had recognized the mistake on their own. They would also proactively address and discuss within the group the consequences that such mistakes could potentially have led to in a clinical situation and what the students could learn from it. One facilitator opined: “Acknowledging one’s mistakes, and addressing them proactively, and thus ensuring patient safety, is crucial in my opinion.” (Mary).

Other facilitators explained how they tended to talk to students about professional standards, mentioning a related procedure or suggesting a guideline rather than directly addressing the mistake, hoping that students would realize their mistakes indirectly. One of the most experienced facilitators said they used to approach feedback by talking about related procedures and guidelines as follows:

So, if team communication is one of the learning objectives and they make a communication mistake, I would focus on communication and ask some questions around that, then perhaps they will realize the mistake themselves. (Jack)

Focusing on specific topics, such as communication as a learning objective and highlighting mistakes in this context, was a facilitating strategy for making students aware of their insufficient performance. Other facilitators described how they usually did not mention students’ mistakes because they assumed the students were aware of them. A facilitator expressed this: “As a rule, they see all the mistakes themselves, they just mention them themselves. I can never mention anything negative because they see it themselves.” (Nancy).

This utterance represents a strong belief in students’ abilities to understand and meaningfully assess their performances in relation to professional standards and guidelines.

## Discussion

In this study, we aimed to explore facilitators’ assumptions about how to use nursing students’ mistakes as learning resources in simulation debriefing. Our discussion focuses on five issues identified in the empirical materials presented above; (1) the facilitators individually assessed what constituted a mistake; (2) they expressed a strong belief about fostering positive feedback to ensure learning; (3) they made an effort to create a safe learning environment; (4) their approaches to addressing mistakes varied; and (5) some of the facilitators assumed that the students were aware of their mistakes. We consider the latter issue to be the most intriguing finding, as it has not been observed in previous studies.

In our interviews, facilitators discussed mistakes as a phenomenon without defining them explicitly, rather than classifying them as actions or behaviours of either “minor” or “severe” characters. Examples of severe mistakes include failure to perform correct procedures, such as incorrect medication handling, and students’ inability to detect patient deterioration. Some facilitators considered actions that could harm or kill the patient as “severe” mistakes, thus underlining the clinical responsibility of performing satisfying professional procedures. Notably, the metaphor of an “elephant in the room” was invoked by facilitators on four occasions during the interviews. According to the Cambridge Academic Content Dictionary [30], an “elephant in the room” refers to a major problem or controversial issue that is blatantly present but avoided as a discussion subject due to discomfort or inconvenience. Their use of this metaphor contextualizes a crucial aspect of facilitators’ experiences by providing feedback in debriefing environments, namely, the need to address obvious, serious mistakes that had been made. At the same time, it highlights the context-dependent nature of addressing mistakes and the delicate, on-the-spot decisions about feedback that facilitators must make.

There is little reason to expect facilitators to be entirely consistent about what constitutes a mistake. For instance, Palominos et al. [7] argue that facilitators often use terms such as failures, errors, and mistakes interchangeably. On a related note, a recent study by Karlgren et al. [31] states that “debriefings have been described as iterating through phases of unawareness of problems, identifying problems, explaining the problems, and suggesting alternative strategies or solutions to manage the problems”. From a clinical perspective, Smith [32] argued that to discuss medical mistakes, it is essential to describe, define, and differentiate them, which aligns with the views of Palominos et al. [7]. By analyzing facilitators’ debriefing interventions, Karlgren et al. [29] identified successful behaviors in using mistakes during simulations as learning opportunities. These facilitators used video recordings of the scenarios to highlight mistakes to students. The facilitators encouraged the students to reflect on what caused the mistakes and how they could prevent similar mistakes in the future. In this way, the facilitators’ interactions contributed to advancing the students’ analysis of their performances.

The facilitators in our study agreed that positive feedback was crucial because they thought that students’ learning increased when they received it. Therefore, they emphasized positive actions regarding whether the students performed successfully. Dai et al. [33] revealed that students found positive feedback to be more credible than negative or corrective feedback, approaches, such as the Learning from Success (LFS) approach to simulation and debriefing, supplementing what has traditionally been described as “learning from mistakes” and the “corrective’ approach” [34].

Dawson et al. [35] analyzed 400 qualitative answers from students who participated in a large survey and found that positive feedback increased student motivation and engagement. Notably, this feedback was related to learning activities other than simulation-based training. They also noted that feedback could be harmful if its affective aspects were ignored during debriefing. However, Hirst et al. [36] discovered that facilitators often provided positive feedback, even when corrective feedback was required. They argued that giving positive feedback only when corrective feedback is necessary can diminish the likelihood of behavioural modification. Similarly, Abelsson and Bisholt [14] found that facilitators who limit themselves to providing positive feedback may inadvertently miss certain learning opportunities. In an interview study with 22 medical simulation facilitators, McQueen et al. [29] found a consensus among participants that providing positive feedback was easy because they liked highlighting positive actions. However, they faced difficulties in identifying mistakes and areas that required improvement. Based on these findings, using students’ mistakes as learning opportunities in a simulation seems challenging for many facilitators. However, facilitators should balance their feedback to encourage students to reflect, identify performance gaps, discuss areas for improvement, and consolidate their knowledge and skills [13].

In line with other research [12, 20, 37], our study found that facilitators are engaged and willing to invest significant effort in establishing a safe learning environment. Moreover, the facilitators in our study considered a safe environment to be beneficial for the learning process because they viewed stress and tension as potential hindrances to learning. Both Topping et al. [10] and Tosterud et al. [23] proposed that the effectiveness of simulation-based training is closely linked to the facilitators’ abilities to foster such safety. However, it is plausible that a certain level of pressure can benefit learning. Tosterud et al. [23] proposed that learning may improve when students are challenged to participate in and actively contribute to simulation-based training. Moreover, they found that this positive pressure can be effectively leveraged when combined with a safe and supportive environment. In such an environment, students may feel more comfortable challenging themselves and are more willing to extend their comfort zones, which can lead to a better understanding [23]. This implies that facilitators must understand how to carefully balance positive stress and reduce negative stress [23].

We conducted interviews with facilitators who, despite their diverse backgrounds, were identified as experienced nurses and simulation facilitators. While we identified some similarities, our analyses revealed significant variations in their approaches to using student mistakes during debriefing. Some facilitators openly discussed students’ mistakes within the group, whereas others preferred to talk about related procedures or guidelines, assuming that students would identify and reflect on their mistakes. A few facilitators chose not to mention mistakes and highlighted what students performed satisfactorily because of their learning objectives and professional expertise. However, research has documented that facilitators often find it challenging to provide corrective and accurate feedback when students make mistakes or underperform during simulations [29, 38]. Some facilitators in our study also experienced tension regarding whether and how to use students’ mistakes as a learning resource. In a seminal case study, Rudolph et al. [39] described such conflicts as “task versus relationship”-dilemmas. These dilemmas present a common challenge for facilitators, involving a value conflict between preserving the students’ sense of safety and mastery on the one hand and addressing mistakes to promote learning about critical issues on the other [40]. In a literature review, Dufrene and Young [41] found that students must be aware of their mistakes to learn from them. Further, they referred to the gold standard guidelines, suggesting that mistakes should be discussed openly in debriefings to achieve learning objectives. Our study problematizes such gold standards for successful learning by exploring facilitators’ assumptions based on the complexity of simulator-based training and professional expertise. However, there seems to be consensus in the simulation literature that facilitators must make students aware of their mistakes [34, 42].

To the best of our knowledge, the observation that a few facilitators in our study opted not to discuss mistakes openly because they assumed that the students were aware of the mistakes, has not been described in existing simulation literature. However, if this assumption is widespread among facilitators, it can have implications for the interactions between facilitators and students during debriefing events. Whether this assumption is widespread among facilitators and its consequences for debriefing practices merit further research.

### Strengths and limitations

One of the strengths of our study is that our informants represented three different universities across Norway. This diversity allows for a more nuanced understanding of the results by incorporating views from various educational settings. The sampling was purposive, ensuring experienced and competent participants from each university. Despite a limited sample size, the interviews yielded rich and varied data. By sampling and interviewing participants from a context in which we had conducted observations of multiple simulation-based training sessions, we could better contextualize valuable information based on their experiences and opinions. Each interview followed the same structure with minor iterative adjustments based on our experiences from preceding interviews. Two of the authors (first and last) were experienced facilitators with prior knowledge and familiarity with the topic. Throughout the research process, the authors held regular meetings to discuss and review every step and to enhance the credibility and trustworthiness of the study.

## Conclusions

Facilitators are central in providing meaningful feedback and supporting students’ learning in simulation-based training. In this study, we described some fundamental assumptions among facilitators regarding how they reflect on what promotes learning during simulator-based training, their accounts of how to promote a safe climate to reflect on students’ performances, and how students’ mistakes can be used as resources for learning during reflective discussions in debriefing. Our study concluded that facilitators’ assumptions about learning affect how they use students’ mistakes as learning opportunities. To gain a better understanding of how facilitators encourage and use students’ mistakes during simulations, future research should explore such interactions in authentic simulation-based training programs.

## Electronic supplementary material

Below is the link to the electronic supplementary material.

Additional file 1: Semi-structured interview guide. The author used the guide during the interviews to gather rich and complementary data

## Data Availability

The dataset used and analyzed during the current study are available from the corresponding author upon reasonable request.
