# Supplementary material for: Turning nursing students’ mistakes into resources for learning in simulation-based training: facilitators’ assumptions about providing feedback in debriefing
Source: BMC Med Educ. 2025 Jan 16;25:76. doi: 10.1186/s12909-024-06628-z (PMC11740578; doi:10.1186/s12909-024-06628-z)
Supplement: Supplementary file 1 — Additional file 1: Semi-structured interview guide. The author used the guide during the interviews to gather rich and complementary data [file 12909_2024_6628_MOESM1_ESM.docx]

Semi-structured interview guide

**Purpose of the interviews**

Extend our knowledge about facilitators' assumptions of providing feedback during debriefing sessions when nursing students make mistakes in simulation-based training.

**Introduction**

Introduction of the study

Information about how the interview will be conducted (how it is structured and how long it will last)

Ask if the participant have any questions before we start the interview

**Demographic data**

Gender

Name

Position/Duration in the position

Experience as a facilitator

Education as a facilitator

Have you been involved in professional groups, projects, development initiatives, or written articles related to simulation work?

As a nursing teacher, how interested are you in simulation-based training? What aspects do you find most engaging?

1. **Experiences with simulation-based training**
2. How often do you facilitate simulation-based training on a regular basis?
3. Do you want to share your thoughts on using simulation-based training as a teaching method?
4. In your opinion, what are the essential qualities and competencies that contribute to the effectiveness of a facilitator, and why do you think they are essential?
5. How can your role as a facilitator influence and shape students' learning experiences, and what specific actions or approaches are most effective in achieving the learning?
6. How do you approach the distribution of roles among students during a simulation to enhance their learning experience, and what strategies or principles guide your decisions?
7. How do you perceive the role of a facilitator during the active execution of a scenario, and what specific actions or behaviours do you believe are crucial for ensuring its success?
8. **Providing feedback when students make mistakes**
9. How do you believe feedback provided during debriefing sessions influences students’ learning and development, and what specific aspects of feedback do you find most impactful?
10. Can you describe your experiences with providing feedback to students, and how do you think this feedback has impacted their learning and development over time?
11. Can you share how you typically respond when students make noticeable mistakes during the simulation sessions and what strategies or approaches you believe are most effective in helping them learn and grow from these experiences?
12. Can you share your experiences by providing honest feedback? What challenges have you encountered in this process, and how have you overcome them?
13. Can you share your experiences handling situations where students are too direct or hurtful when giving feedback to their peers? What strategies do you use to ensure that feedback remains constructive and respectful?
14. **Use of structured frameworks during the debriefing**
15. How do you feel about using structured frameworks during debriefing sessions, and what impact do you believe they have on the effectiveness of the debriefing process?
16. When facilitating, how do you use the framework? Do you follow it step-by-step, or do you adapt it based on the situation?
17. What potential disadvantages might arise from using structured frameworks?
18. **The atmosphere in the debriefing room**
19. What strategies do you use as a facilitator to create and maintain a positive and safe atmosphere during simulation debriefings?
20. What are your thoughts on using narratives or real-life stories in debriefing sessions?

What impact do you think they have?

1. How do you manage varying student behaviours during simulation debriefings, such as inactivity, disengagement, excessive laughter, or nervousness?
2. What techniques do you use to reduce tension and foster a relaxed atmosphere within the group?
3. What responsibilities do you believe students should take on during simulation activities?

**Summing up**

Is there anything else you want to add regarding this interview?

Thank you for participating in the study
